# NF-κB is involved in the regulation of autophagy in mutant p53 cells in response to ionizing radiation

**DOI:** 10.1038/s41420-021-00533-w

**Published:** 2021-06-25

**Authors:** Ying Zhu, Wenqing Zuo, Xiao Shen, Yali Liu, Yifan Zhao, Yajie Xiong, Huimin Cao, Yan Wang, Zhongqin Liang

**Affiliations:** 1 Department of Pharmacology, College of Pharmaceutical Sciences, Soochow University, Suzhou, China; 2 Department of Pharmacy, Suzhou Kowloon Hospital, Shanghai Jiaotong University School of Medicine, Suzhou, China

**Keywords:** Cancer, Autophagy

## Abstract

Chemotherapy and ionizing radiation (IR) can induce autophagy in tumor cells. Here, we report that the level of autophagy in tumor cells was related to the background of *p53* gene that NF-κB acts as a negative regulator of autophagy in mutant p53 (p53-R273H) cells, and that acetylation was involved in the IR-induced nuclear translocation of NF-κB. We found that autophagy-related proteins were highly expressed in wild-type p53 (wt-p53) cells and that IR increased their levels further. p53-R273H cells exhibited low levels of autophagy; there was no change following IR treatment. The nuclear translocation of p65 was upregulated in p53-R273H cells following IR; when p65 was competitively inhibited from entering the nucleus with SN50, the level of autophagy increased. The nuclear translocation of p65 was mediated by p300; this factor also regulates the nuclear behavior of NF-κB. The knockdown of p300 in p53-R273H cells led to an inhibition of p65 expression and an increase in autophagy. In addition, the inhibition of p300 or p65 not only activated autophagy, it also induced radiosensitivity in p53-R273H cells. The relationship between the *p53* gene, NF-κB, and autophagy was further analyzed in a mouse model of xenograft tumors and in clinical tumor pathological specimens; the results were consistent with the in vitro experiments. Our findings indicate that autophagy may be regulated by NF-κB in p53-R273H cells. These findings may help to improve the therapeutic strategy adopted for tumors related to the mutant *p53-R273H* gene; such therapy would aim to target NF-κB to induce autophagy.

## Introduction

Autophagy is a process in which cells use lysosomes to degrade their damaged organelles and macromolecules. This represents an important regulatory mechanism for cell growth, differentiation, maturation, and death, but is also related to a variety of human diseases, including tumors [1]. Many studies have shown that there is a certain crossover between autophagy and apoptosis; however, compared with apoptosis, tumor cells are less tolerant to the death pathway associated with autophagy.

Researchers have previously shown that ionizing radiation (IR) can induce autophagy [2], although there is significant debate relating to the significance of autophagy in tumors experiencing, including contradictory results. For example, Apel et al. used siRNA technology to inhibit autophagy-related proteins, and this resulted in a variety of tumor cells becoming increasingly sensitive to radiotherapy [3]. However, contradictory results were obtained by Daido et al.; these authors used DNA-PK inhibitors to activate autophagy in glioma cells, thus increasing the radiosensitivity of glioma cells [4]. Previous studies in our laboratory have also shown that activation of autophagy increases the sensitivity of glioma stem cells to IR [5]. Therefore, it is still unclear whether autophagy should be induced or suppressed when treating tumors.

Research suggests that the role of autophagy in cancer cells depends upon certain factors, such as cell type, the specific characteristics of tumor cells, the microenvironment of the tumor, and the type of treatment applied. In addition, a variety of radiation-resistant molecules, such as PI3K/AKT, EGFR, NF-κB, and p53, may play an important role in the regulation of autophagy, thus indicating that the mechanisms that regulate autophagy are very complex. Many questions and contradictory findings have yet to be clarified.

The *p53* gene is known to act as a tumor suppressor gene; the expression levels of this gene are extremely low in normal cells. However, over 50% of malignant tumors are known to express *p53* mutations or deletions. Studies have found that mutations in the *p53* gene can stimulate the mTOR signaling pathway by inhibiting certain proteins and enzymes related to autophagy and thus inhibit the formation of autophagy vesicles or their fusion with lysosomes [6, 7]. In addition to the status of the *p53* gene, the localization of the p53 protein may also exert differential effects on autophagy. It is generally believed that the induction of autophagy occurs when the p53 protein is located inside the nucleus. However, autophagy is inhibited when the p53 protein is located in the cytoplasm [6, 8]. It is therefore evident that the regulatory mechanism of p53 with regard to autophagy is quite complex and may depend upon several factors, including the status of the *p53* gene, the microenvironment in which cells are located, the pressure the cells are under, and their subcellular localization [8, 9].

NF-κB, as an important transcription factor, can directly influence the occurrence and development of tumors, and can also regulate the numbers of signaling pathways including those involved in the cell cycle, apoptosis, and autophagy [10]. Previous studies have found that mutant forms of the p53 protein (mut-p53) can interact directly with NF-κB p50 to form an NF-κB p50/mut-p53 protein complex that can then bind to the promoter of the autophagy gene *Atg12* to play an inhibitory role on autophagy [11]. These findings suggest that there is a certain relationship between these factors that may be useful from a clinical point of view. Many studies have reported the interaction between p53 and NF-κB. The wild-type p53 has often been shown to inhibit the activation of NF-κB; however, in some cases, wild-type p53 can also work with NF-κB in response to more intense stress stimuli [12–14].

It has recently been reported that NF-κB can regulate the transcriptional activity of the *p53* gene and that p53 can regulate the activity of NF-κB [15]. For example, Vaughan et al. reported that p53 can mediate the significant upregulation of NF-κB, thus forming a strong pro-cancer effect [16]. In many cancer cells, lowering the level of the p53 mutant leads to the loss of the gain-of- function (GOF) phenotype, a process that is directly associated with p53 mutation. However, when RNAi is used to interfere with NF-κB, the GOF phenomenon in cancer cell lines is reduced, suggesting the existence of a complex relationship between p53 and NF-κB [17, 18]. In a previous study, Cooks et al. demonstrated the extensive accumulation of mutant p53 protein in inflammatory colon cancer glands and that this was associated with NF-κB activation and sustained DNA damage [19]; these findings further verified the fact that mutant p53 can promote the activation of NF-κB. In addition, Rahnamoun et al. described several mechanisms by which wild-type p53 and mutant p53 interact with NF-κB [20]. These authors believed that the GOF associated with mutant p53 can often involve gene regulation and there was an overlap between mutant p53 and NF-κB. Furthermore, these researchers considered that only mutant p53 can bind to TNF promoters to participate in the maintenance or enhancement of effects in the TNF signaling pathway that promote the expression of immune genes [21]. Further research showed that, unlike wild-type p53, most mutant p53 proteins interact with other transcriptional regulators (such as NF-κB and NF-Y) to regulate gene expression [20].

In addition, acetylation is also known to play an important role in the functional activities of p53, NF-κB, and autophagy. P300 is an important histone acetyltransferase; this enzyme not only regulates histone activity and promotes gene transcription activation *via* acetylation, it can also directly acetylate some nonhistone transcription factors, such as p65 and p53, thus exerting influence on cell growth and differentiation. In a previous study, Gu et al. discovered that p53 can be acetylated, making it the first nonhistone protein to be acetylated in this manner [22]. When DNA damage occurs, p300 can acetylate and activate *p53*, the tumor suppressor gene. Acetylation increases the stability of the p53 protein, thus promoting its binding activity with other proteins; this also improves antiviral activity [23]. Studies have shown that p300 protein is a coactivator of p53. When DNA damage occurs, the NF-Y complex can bind to the CCAAT box of the *p53* gene; the status of the *p53* gene may determine the identity of other members of the complex. In the presence of mutant p53, p300 will be recruited first, leading to histone acetylation and an overall increase in the extent of acetylation on the promoter; this leads to abnormalities in the regulation of the cell cycle [24]. The p65 subunit of NF-κB also contains sites that can be acetylated by p300; these sites play an important role in regulating the nuclear behavior of NF-κB and can also be involved in the regulation of autophagy when NF-κB is transferred into the nucleus. In addition, Wan et al. confirmed that mTORC1 phosphorylates acetyltransferase p300, thereby regulating autophagy [25]. Thus, it is evident that there is a regulatory effect between acetylation and p53, NF-κB, and autophagy. However, until now, few studies have investigated the specific relationship between acetylation and p53, NF-κB, and autophagy.

Therefore, the purpose of this study was to elucidate the effect of mutant p53 on autophagy, verify the regulatory effect of NF-κB on autophagy during IR treatment, and identify the relationships between autophagy, p53, and NF-κB from the perspective of acetylation.

## Results

### The expression of autophagy-related proteins was related to the status of the *p53* gene

Preliminary CCK8 assay results showed that the survival of the two glioma cell lines (U251 and U87) differed significantly following IR. U87 cells were clearly sensitive to IR; however, U251 cells were less sensitive to IR and exhibited a strong survival rate that did not change significantly with the dose of IR (Fig. S1A). Flow experiments showed that the extent of apoptosis in U251 cells was not as obvious as that in U87 cells and that the rate of survival was also higher in U251 cells (Fig. S1B). Colony-formation assays showed that U251 cells exhibited poor sensitivity to IR (Fig. S1C). These results showed that the inhibitory effect of IR on wild-type p53 cells was more obvious than that on mutant p53-R273H cells.

**Fig. 1:**
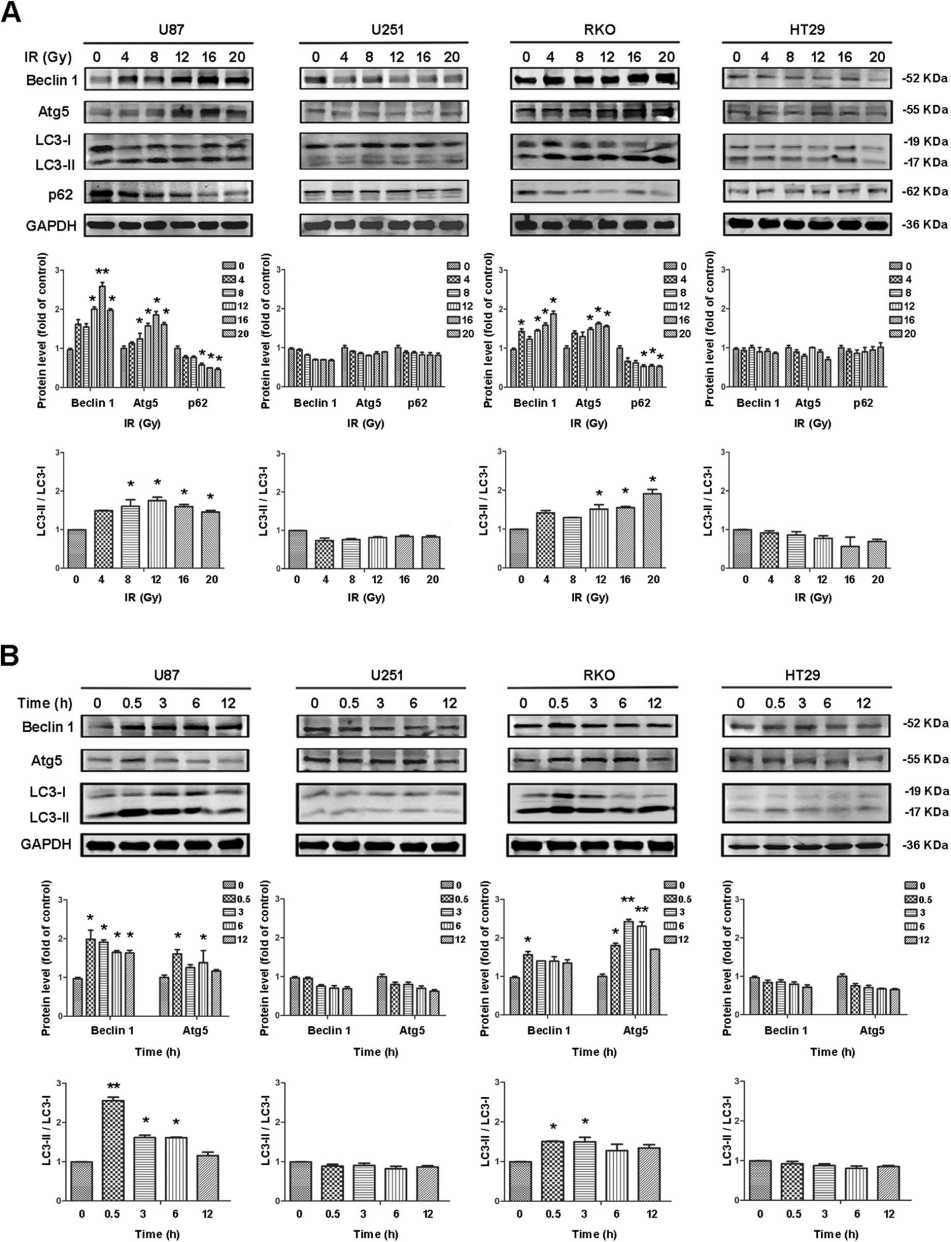
The expression of autophagy-related proteins was related to the status of the *p53* gene. **A** Western blotting of Beclin 1, Atg5, LC3, and p62 proteins at different IR doses (0.5 h) in U87, U251, RKO, and HT29 cells. **B** Western blotting analysis of Beclin 1, Atg5, and LC3 proteins at different time points after IR (12 Gy) in U87, U251, RKO, and HT29 cells (^*^P < 0.05, ^**^P < 0.01 vs. control).

Since autophagy mediates the survival of cells, we used western blotting to detect the expression of autophagy-related proteins in each cell line, and found that the levels of Beclin 1, Atg5, and LC3 proteins gradually increased with increasing dose of IR in both U87 and RKO (wt-p53) cells. Accordingly, levels of the autophagy substrate p62 protein decreased as the extent of autophagy increased. However, there was no significant change in the levels of autophagy-related proteins in U251 and HT29 (p53-R273H) cells (Fig. 1A). At the same radiation dose, the levels of Beclin 1, Atg5, and LC3 in U87 and RKO cells increased with time after IR, the most obvious increase was at 0.5 h. Changes in the levels of autophagy-related proteins in U251 and HT29 cells were not as obvious (Fig. 1B), thus indicating that autophagy was inhibited in the mutant p53-R273H cells.

### The level of LC3 may be associated with NF-κB activation after IR

**Fig. 2:**
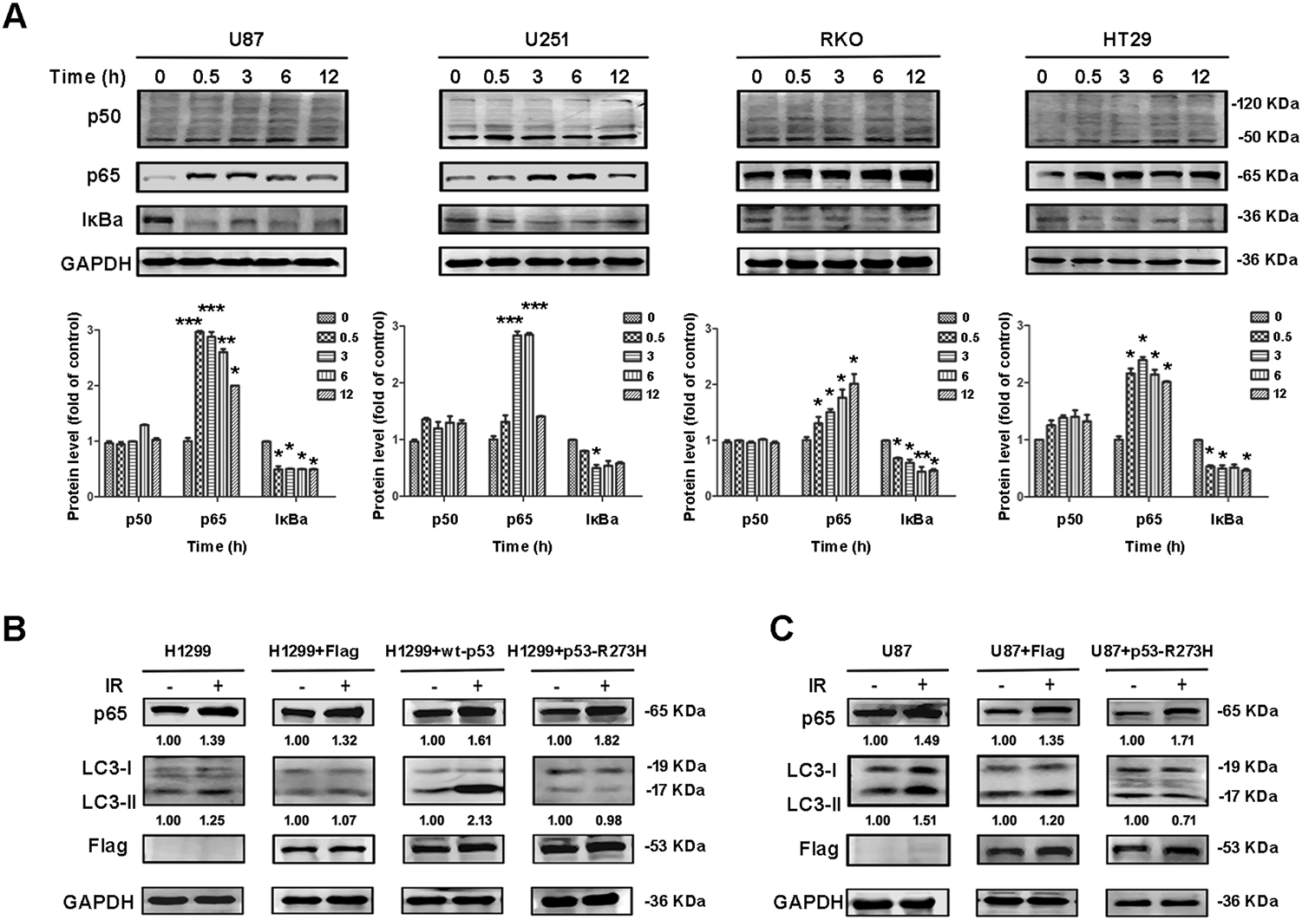
The level of LC3 may be associated with NF-κB activation after IR. **A** Western blotting analysis of the levels of p50, p65, and IκBa proteins at different time points after IR (12 Gy) in U87, U251, RKO, and HT29 cells. **B** After transfecting with Flag, wild-type p53, and mutant p53-R273H plasmids in p53-null H1299 cells, western blotting was used to analyze the p65 and LC3 protein levels after IR. **C** U87 cells were transfected with Flag, mutant p53-R273H plasmids, and the protein changes of p65 and LC3 were analyzed by westeinterference chains, then western blottingrn blotting after IR (^*^P < 0.05, ^**^P < 0.01, ^***^P < 0.001 vs. control).

It has been reported that mutant p53 interacts with a variety of cytokines, such as NF-κB. Furthermore, p53 is known to interact with NF-κB to regulate the survival of tumor cells. Therefore, we measured the levels of NF-κB and IκBa at different time points after IR in different cell types. Typically, NF-κB is a dimer consisting mainly of p50 and p65 subunits. Therefore, we investigated changes in the levels of both p50 and p65 proteins. We found that over time, the levels of p65 increased in all four cell types; the levels of IκBa decreased and there were no significant changes in the levels of p50 (Fig. 2A). To further verify the relationship between autophagy and NF-κB/p53 after IR, we also transfected wild and mutant p53-R273H plasmids into H1299 (null p53) cells. Analysis showed that cells transfected with wild-type p53 plasmids exhibited increased levels of autophagy after IR, while cells transfected with mutant p53-R273H plasmids showed no significant changes in autophagy after IR, except for an increase in the levels of NF-κB (Fig. 2B). We also transfected U87 (wild-type p53) cells with mutant p53-R273H plasmids and observed no significant changes in terms of autophagy after IR (Fig. 2C). This proved that the extent of autophagy after IR was related to NF-κB and the status of the *p53* gene.

**Table 1:** The chain of interference for transfection cells.

The sequence of siRNA		
siRNA	Base sequence	
Negative control (NC)	Sense	5′-UUCUCCGAACGUGUCACGUTT-3′
	Antisense	5′-ACGUGACACGUUCGGAGAATT-3′
si-p300^728^ (#1)	Sense	5′-GUCCUGGAUUAGGUUUGAUTT-3′
	Antisense	5′-AUCAAACCUAAUCCAGGACTT-3′
si-p300^1807^ (#2)	Sense	5′-GGACUACCCUAUCAAGUAATT-3′
	Antisense	5′-UUACUUGAUAGGGUAGUCCTT-3′
si-p300^3797^ (#3)	Sense	5′-CAUCACGGGUAUACAAAUATT-3′
	Antisense	5′-UAUUUGUAUACCCGUGAUGTT-3′
si-Atg5^486^ (#1)	Sense	5′-GACGUUGGUAACUGACAAATT-3′
	Antisense	5′-UUUGUCAGUUACCAACGUCTT-3′
si-Atg5^695^ (#2)	Sense	5′-GUCCAUCUAAGGAUGCAAUTT-3′
	Antisense	5′-AUUGCAUCCUUAGAUGGACTT-3′
si-Atg5^938^ (#3)	Sense	5′-GACCUUUCAUUCAGAAGCUTT-3′
	Antisense	5′-AGCUUCUGAAUGAAAGGUCTT-3′

### Acetylation was involved in the IR-induced nuclear translocation of NF-κB

**Fig. 3:**
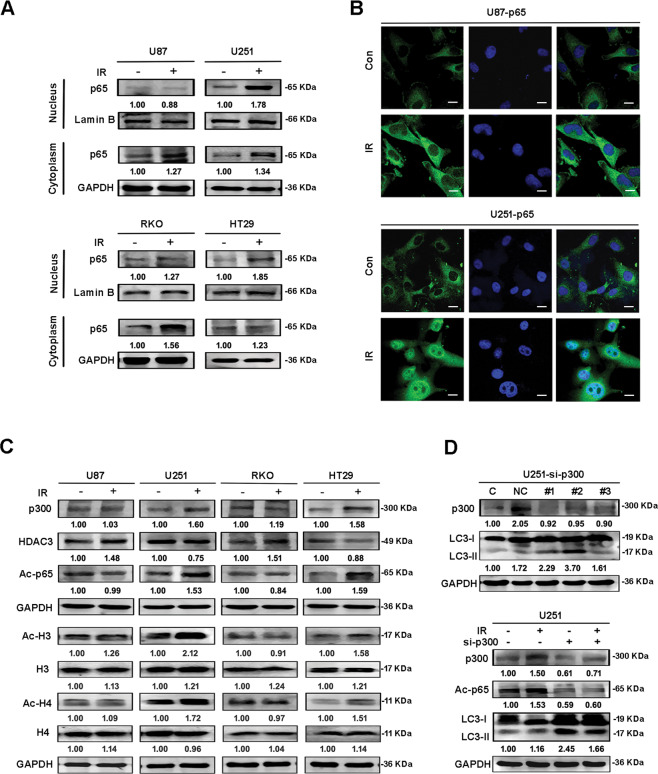
Acetylation was involved in the IR-induced nuclear translocation of NF-κB. **A** Samples were collected with the nucleus and cytoplasm separation kit, and then western blotting was used to analyze the changes of p65 protein in the nucleus and cytoplasm after IR in U87, U251, RKO, and HT29 cells. **B** Immunofluorescence further verified the localization changes of p65 in U87 and U251 cells before and after IR (×630). **C** Western blotting was used to analyze the changes of p300, Ac-p65, HDAC3, Ac-H3, H3, Ac-H4, and H4 proteins after IR in the cell lines. **D** P300 and LC3 protein levels were detected by interfering with p300 in U251 cells with interference chains, then western blotting was used to analyze the changes of p300, Ac-p65, and LC3 proteins by 12 h after the interference of p300 in U251 cells. Table 1.

In nonstimulated cells, NF-κB is mostly located in the cytoplasm and exists in an inactive status. When stimulated, NF-κB moves into the nucleus and adopts a regulatory role [26]. In the present study, we found that the nuclear translocation of p65 was not obvious, but there was an obvious increase of p65 in the cytoplasm of U87 and RKO cells after IR. However, p65 was significantly transferred from cytoplasm to nucleus in U251 and HT29 cells (containing mutant p53-R273H) (Fig. 3A). The results arising from immunofluorescence experiments were consistent with those arising from western blotting (Fig. 3B).

Next, we considered the identity of the specific mechanism that is responsible for the differences in p65 nuclear translocation between wild and mutant p53 cells after IR. It has been reported that acetylation at different sites within p65 can regulate the nuclear function of NF-κB and that p300 is involved in this process [27]. P300 can regulate gene expression *via* the acetylation of histones and can also directly acetylate some nonhistone transcription factors, such as p65. In addition to acetylation, histone deacetylase HDAC3 may also be involved in the deacetylation of NF-κB [28]. Therefore, we further detected the levels of p300 and HDAC3 proteins in cells with a different p53 status before and after IR. We found that the levels of p300 and Acetyl-NF-κB-p65 (Ac-p65) in U251 and HT29 cells increased after IR, while the levels of deacetylase HDAC3 decreased slightly. However, the levels of p300 and Ac-p65 did not change significantly in U87 and RKO cells, although the levels of HDAC3 increased (Fig. 3C). Accordingly, we found that the level of autophagy in U251 cells increased when the levels of p300 were changed, while the levels of Ac-p65 decreased (Fig. 3D). Collectively, these results indicated that an increase in the extent of acetylation may be related to the nuclear translocation of NF-κB after IR.

### Evidence for NF-κB as a negative regulator of autophagy in mut-p53 cells after IR

**Fig. 4:**
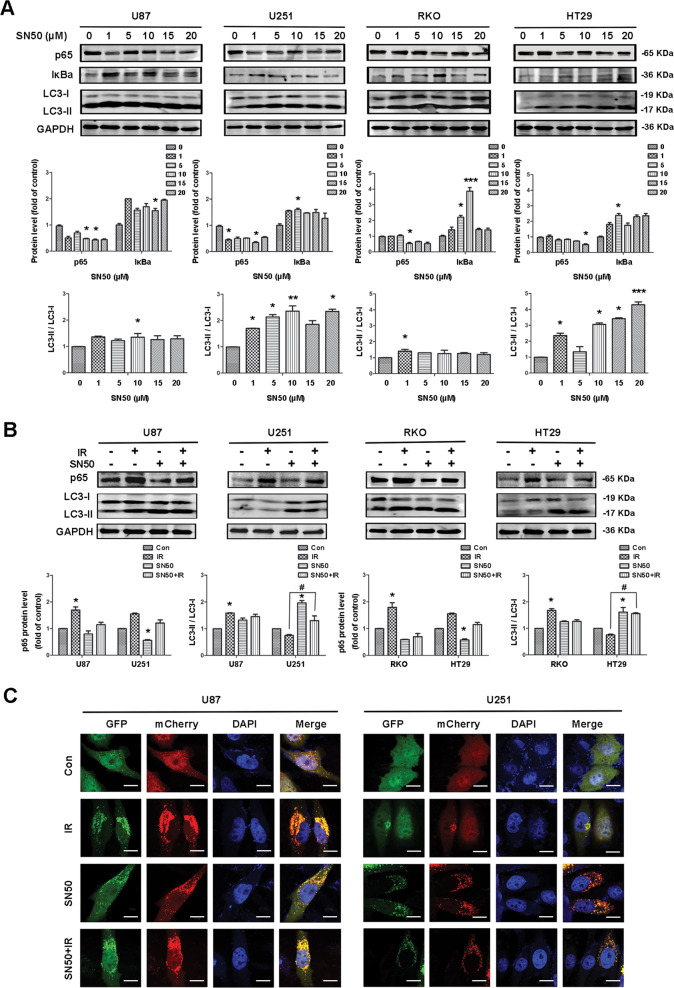
Evidence for NF-κB as a negative regulator of autophagy in mut-p53 cells after IR. **A** Western blotting was used to analyze the changes of p65, IκBa, and LC3 proteins after treatment with SN50 at different concentrations in U87, U251, RKO, and HT29 cells. **B** Western blotting was used to analyze the changes of p65 and LC3 proteins with SN50 pretreatment before IR in U87, U251, RKO, and HT29 cells. **C** U87 and U251 cells were transfected with GFP-mCherry-LC3 plasmid, then treated as required in each group; finally images were taken by confocal immunofluorescence (×630) (^*^P < 0.05, ^**^P < 0.01, ^***^P < 0.001 vs. control; ^*#*^*P* < 0.05 compared to the IR group).

Previous experimental results showed that NF-κB activity was related to autophagy and that the translocation of NF-κB into the nucleus inhibited autophagic activity. Next, we considered whether the inhibition of NF-κB would promote autophagy. We treated cells with SN50, a protein that inhibits the entry of p65 into the nucleus. We observed a reduction of NF-κB and a significant increase in the level of autophagy in U251 and HT29 cells, thus indicating that the inhibition of NF-κB in the nucleus can activate autophagy (Fig. 4A). When pretreated with SN50 before IR, the levels of autophagy in both U251 and HT29 cells were significantly higher than those treated with IR alone (Fig. 4B).

In order to further demonstrate the increased levels of autophagy following the treatment of U251 cells with SN50, we transfected U87 and U251 cells with the fluorescent GFP-mCherry-LC3 plasmid. Analysis showed that flow of autophagy was relatively unobstructed in U87 cells. Following IR treatment, there was a significant increase in fluorescence, particularly with regard to red fluorescence, thus indicating increased levels of autophagy. However, U251 cells exhibited a different trend from U87 cells. Analysis showed that autophagy was not obvious in the control group of U251 cells; the flow of autophagy was unobstructed and there was no obvious change following IR treatment. However, after SN50 treatment, there was a significant increase in autophagy; the flow of autophagy changed from being blocked to unobstructed. The increase in the level of autophagy in cells pretreated with SN50 prior to IR treatment was also significantly higher than that in the IR group (Fig. 4C). These results suggested that SN50 promoted autophagy by inhibiting the nuclear entry of NF-κB.

**Fig. 5:**
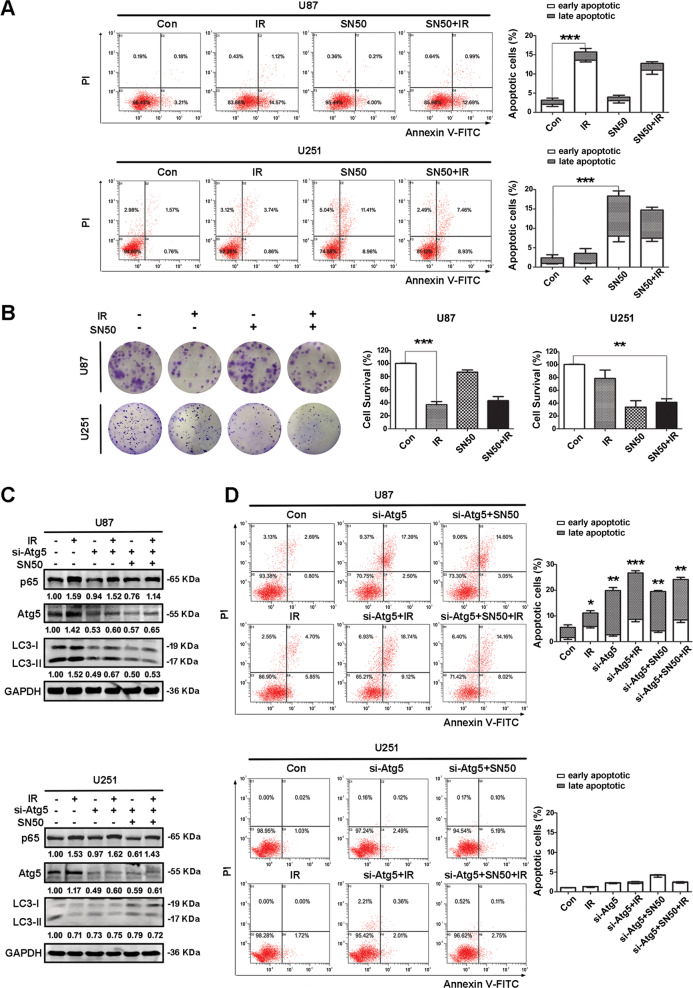
Evidence for NF-κB as a negative regulator of autophagy in mut-p53 cells after IR. **A** U87 and U251 cells were treated according to different requirements, then Annexin V-FITC/PI double dyes were used to stain the cells and determine cell apoptosis 24 h later in each group. **B** U87 and U251 cells were treated as required, and colony-formation assay detected the cell survival. **C** Inhibiting the expression of autophagy-related protein Atg5 by siRNA, then western blotting was used to analyze the changes of p65, Atg5, and LC3 proteins in each group. **D** Inhibiting the expression of autophagy-related protein Atg5 by siRNA, then Annexin V-FITC/PI double dyes were used to stain the cells and determine cell apoptosis 24 h later in each group (^*^P < 0.05, ^**^P < 0.01, ^***^P < 0.001 vs. control). Table 1.

Next, we considered the effects of enhanced autophagy on cell survival. Annexin V-FITC/PI results revealed a significant increase in the levels of autophagy and apoptosis after IR, although changes were not obvious in U87 cells following SN50 treatment. In contrast, U251 cells showed no significant changes in autophagy after IR, and did show increased levels of autophagy and apoptosis after SN50 treatment (Fig. 5A). Colony-formation assays showed that cell proliferation decreased after IR treatment in U87 cells and decreased after SN50 treatment in U251 cells (Fig. 5B). However, after inhibiting the expression of autophagy-related protein Atg5 by siRNA, there was no significant change in the level of apoptosis following SN50 and IR treatment in U251 cells (Fig. 5C, D). This also indicated that autophagy had an important relationship with NF-κB, and the effect of SN50 treatment was not obvious after autophagy inhibition.

### Analysis of the correlation between NF-κB and autophagy with mutant p53 in vivo

**Fig. 6:**
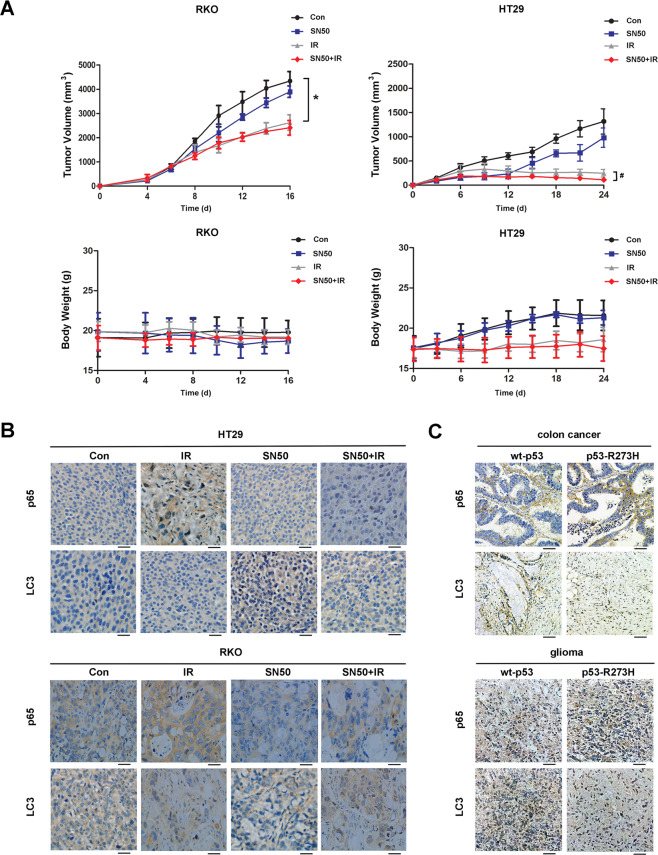
Analysis of the correlation between NF-κB and autophagy with mutant p53 in vivo. Tumor models were established for subcutaneous transplantation of RKO and HT29 cells, and local X-ray radiation treatment was performed as described in the “Materials and methods” after tumor formation. **A** The tumor size was measured using a Vernier caliper and body weight was determined using an electronic balance. **B** Immunohistochemical method was used to detect the changes of p65 and LC3 before and after IR in RKO and HT29 tumor models (×400). **C** Immunohistochemical method was used to detect the protein levels of p65 and LC3 in each tumor sample (×400) (^*^P < 0.05 vs. control; ^*#*^*P* < 0.05 compared to the IR group).

Next, we used HT29 (p53-R273H) and RKO (wt-p53) cell lines to establish an in vivo mouse xenograft tumor model. Once established, we monitored tumor and body weight. There were no significant changes in tumor volume when compared between the IR group and the SN50 + IR group in RKO mice (Fig. 6A). However, in HT29 mice, we found that the tumor volume of the IR + SN50 group was smaller than that of the IR group, indicating that SN50 can inhibit tumor growth in cases involving p53-R273H mutation. Immunohistochemical results also showed that autophagy increased after SN50 treatment in HT29 mice (Fig. 6B); these findings were consistent with our in vitro experiments.

To further verify the relationship between NF-κB and LC3, we extracted DNA from clinical tumor samples and detected the expression levels of the *p53* gene by sequencing. Samples with the wild-*p53* gene and mutant *p53-R273H* gene were selected for experiments. Immunohistochemical results showed that tumor samples expressing the *p53-R273H* gene had higher levels of NF-κB and lower levels of autophagy (Fig. 6C). Consequently, p53-R273H mutation had an inhibitory effect on autophagy.

## Discussion

In this study, we found that the expression levels of the *p53* gene can influence the basic levels of autophagy in cells, and that mutation in the *p53* gene mediates the regulation of autophagy by IR through NF-κB and the process of acetylation.

Previous studies in our laboratory discovered that neural stem cells have high basal levels of autophagic activity [29], while glioma stem cells have low basal levels of autophagic activity [5]. Following the activation of autophagy, glioma stem cells showed increased sensitivity to IR, indicating that there was a specific relationship between autophagic activity and radiosensitivity [5]. In recent years, we also identified significant differences in the levels of autophagy in cells with different *p53* gene backgrounds; these differences directly affected the sensitivity of cells to IR. In the present study, we found that cells expressing wild-type p53 showed high levels of basal autophagy; however, the basal levels of autophagy in cells expressing mutant p53-R273H were very low; the induction of autophagy in these cells increased radiosensitivity (Fig. 5). It is known that the regulation mechanism of p53 and autophagy is quite intricate, which may be related to the status of the *p53* gene, and subcellular localization of p53 proteins. Morselli et al. demonstrated that mutants constantly characterized by a cytoplasmic distribution (e.g., p53E258K, p53R273H, and p53R273L) were highly efficient autophagy inhibitors, which is also consistent with our results [30]. This suggested that mutant p53 may be related to autophagy after IR, but the regulatory mechanism involved still needs to be further studied.

Wild-type p53, as a tumor suppressor, usually inhibits the activation of NF-κB [12, 31]. However, Cooks et al. reported that mutant p53 could promote the activation of NF-κB [19]. In contrast, other studies have suggested that NF-κB can activate mutant p53. In this study, we examined the effects of IR on the p50 and p65 subunits of NF-κB, IκBa, and LC3, in four different types of tumor cells with different *p53* gene backgrounds. Over time, p65 increased and IκBa decreased in several cell lines. Furthermore, the results of nuclear/cytoplasmic separation and immunofluorescence experiments showed that p65 increased significantly in the cytoplasm but did not lead to significant changes in expression within the nuclei of U87 and RΚO cells (wild-type p53) after IR. In contrast, mutant p53-R273H cells (U251 and HT29) showed significant nuclear translocation of p65 (Fig. 3).

It has been reported that acetylation plays an important role in regulating the nuclear behavior of NF-κB. The p65 subunit of NF-κB can be acetylated at several different sites. The acetylation of the lysine residues of NF-κB regulates various functions of NF-κB, including transcriptional activation, DNA binding, and binding to its inhibitor IκBa [28]. As an important acetyltransferase, p300 does not only regulate gene expression by acetylating histones, it also directly acetylates some nonhistone transcription factors, such as p65 and p53, and therefore plays an important role in life activities. Five sites on the p65 subunit of NF-κB are known to be acetylated by p300, including lysine 122, 123, 218, 221, and 310 [27, 32]. The acetylation of lysine 310 is necessary for the complete activation of the NF-κB complex; acetylation of lysine 221 enhances p65 binding to the κB enhancer, and acetylation of lysine 221, alone or in combination with lysine 218, destroys the assembly of p65 with IκBa. In addition to acetylation, the histone deacetylase HDAC3 may also be involved in the deacetylation of NF-κB. This form of deacetylation enhances the binding of NF-κB to IκBa, thus leading to the IκBa-dependent-translocation of the NF-κB complex [28]. It has been reported that the expression of HDAC3 can inhibit the activation of NF-κB after TNFa stimulation, while HDAC1, 2, 4, 5, and 6 have no such effect [33]. Thus, acetylation and deacetylation play different regulatory roles in the various biological functions of NF-κB.

Our present research also detected changes in the levels of p300, Ac-p65, and HDAC3 before and after IR in different cell types. We found that levels of p300 and Ac-p65 increased, while the levels of HDAC3 decreased after IR in both U251 and HT29 cells. In U87 and RΚO cells, the levels of p300 and Ac-p65 did not change significantly; however, the levels of HDAC3 increased. When the expression of p300 was disturbed by siRNA in U251 cells, we found that there was a reduction in the levels of Ac-p65 and an increase in autophagy. These results showed that NF-κB activity is associated with autophagy and that the nuclear translocation of NF-κB inhibits autophagic activity. To verify whether the inhibition of NF-κB facilitates autophagic activation, we treated cells with the NF-κB competitive inhibitor SN50 and found that as the expression of NF-κB decreased, there was an increase in autophagy in U251 and HT29 cells; no such changes occurred in U87 and RKO cells. We transfected the fluorescent GFP-mCherry-LC3 plasmid to further investigate the changes of autophagy in U87 and U251 cells. Immunofluorescence data showed that autophagy was unobstructed in U87 cells after IR treatment; the fluorescence increased significantly, indicating a significant increase in autophagy. However, SN50 alone, or combined with IR treatment, had no significant effect on autophagy. In contrast, U251 cells exhibited changes that differed from those seen in U87 cells. Data showed that autophagy was not unobstructed in U251 cells, and there was no change after IR treatment. However, after SN50 treatment or combined IR treatment, there was a significant increase in autophagy (Fig. 4). These results indicated that the inhibition of NF-κB-promoted autophagy may be associated with the *p53* gene.

Flow cytometry showed that the extent of apoptosis was not significant following SN50 treatment in U87 cells. However, U251 cells showed increased levels of apoptosis and reduced proliferation after SN50 treatment, further indicating that the inhibition of NF-κB can promote autophagy, induce apoptosis, and inhibit proliferation, in mutant p53 cells. The results of our in vivo experiments further verified the conclusion of our in vitro experiments. Similarly, our clinical samples exhibited higher levels of NF-κB and lower levels of autophagy in mutant p53-R273H samples compared with wild-type p53 samples (Fig. 6), thus indicating that there was a specific relationship between these factors.

In conclusion, our current research demonstrated that autophagy is associated with the *p53* gene. IR can inhibit autophagy and promote cell survival by promoting the acetylation of NF-κB in the nucleus of mutant p53-R273H cells. Therefore, this study identified the relationship between *p53* gene status and autophagy and further demonstrated the significance of autophagy activation in the treatment of tumors.

## Materials and methods

### Cell lines and culture

Non-small-cell lung cancer cell line H1299, glioma cell lines U87 and U251, and colon cancer cell lines RKO and HT29 were purchased from the Type Culture Collection of the Chinese Academy of Sciences (Shanghai, China). The cells were cultured in DMEM/High glucose medium (HyClone, Los Angeles, USA) supplemented with 10% fetal bovine serum (Gibco, United States, California) and cultured at 37°C in a humidified atmosphere containing 5% CO_2_.

### Patients and tumor specimens

Human tissue samples were collected from surgically resected specimens from patients in the Suzhou Kowloon Hospital (glioma tissues, *n* = 12, Suzhou, China) and Affiliated Hospital of Jiangsu University (colon cancer tissues, *n* = 10, Zhenjiang, China) with written informed consent of patients.

### IR condition, antibodies, and reagents

The cells were irradiated with Rad Source biological X-ray irradiance (RS-2000 Pro, Rad source, Inc) under the condition of vertical X-ray irradiation, dose rate of 1.5 Gy/min, and total dose of 12 Gy. The antibodies of Beclin 1, Atg5, p62, p50, p65, IκBa, HDAC3, Flag, and GAPDH were purchased from Cell Signaling Technology (Massachusetts, USA). LC3 was purchased from Novus Biologicals (Littleton, Co, USA). P300 was purchased from Bethyl Laboratories (Montgomery, TX, USA). Lamin B was obtained from MultiSciences Biotech (Hangzhou, China). SN50 was purchased from Med Chem Express (Monmouth Junction, NJ, USA).

### Cell Counting Kit-8 (CCK-8)

Cell viability was measured using the CCK-8 (Beyotime, Jiangsu, China). First, the cells were seeded into 96-well plates at a rate of 2 × 10^3^ cells/well during the exponential phase of growth and allowed to adhere overnight. After specific treatment, 10 μL CCK-8 solutions were added to each well and the plate was incubated at 37 °C for 1–4 h. The OD value for each well at 450 nm wavelength was measured using a microplate reader. The assay was repeated three times.

### Apoptosis assay

Cell apoptosis was assessed by an Annexin V-fluorescein isothiocyanate (FITC)/propidium iodide (PI) apoptosis detection kit (Beyotime, Jiangsu, China). The cells were cultured in 6-well plates (NEST Biotechnology Co. LTD. Wuxi, China) for 24 h and then treated as required. Cells were collected 24 h after treatment, and resuspended with 500 uL of binding buffer, and then incubated with 5 μL of Annexin V-FITC and 10 μL of PI at room temperature for 15 min in the dark. At the end, apoptosis was analyzed by LSRII flow cytometry and FACSDiva software.

### Colony-formation assay

Quantitative cells were placed in 6-well plates overnight and then treated as required. The cells were cultured for 14 d and stained with crystal violet dye (Beyotime, Jiangsu, China). The number of visible colonies (>50 cells) was observed and counted by a microscope.

### Western blotting

Cells were harvested followed by cracking with cold lysis buffer. Cell lysates was collected by centrifugation and protein concentration was measured by BCA Protein Assay Kit (Thermo Scientific, Rockford, USA). Proteins of each sample were separated on sodium dodecyl sulfate−polyacrylamide gel electrophoresis (SDS-PAGE) and then transferred onto polyvinylidene difluoride membranes. The blots were blocked with 5% nonfat milk and then incubated with the primary antibodies at 4 °C overnight, followed by incubation with secondary antibodies at room temperature for 1 h in the dark. The blots were detected by Odyssey Infrared Imaging System (Li-COR Biosciences, Lincoln, NE, USA) and Image J software was used to quantify the data.

### siRNA and plasmid transfection

siRNA for Atg5 and negative control siRNA were purchased from GenePharma (GenePharma, Shanghai, China). P300, mutant p53-type, and wild p53-type plasmids were purchased from Suzhou Golden Wisdom Biological Technology Co., Ltd. Cells were cultured in 6-well plates for 24 h and transfected with either siRNA or plasmids using lipofectamine 3000 (Invitrogen) according to the manufacturer’s protocol.

### Immunofluorescence staining

The cells were plated into 12-well plates and then treated as required. After 24 h of culture, cells were fixed with 4% paraformaldehyde for 10 min at 4 °C, washed with PBS, and permeabilized with 0.1% Triton X-100 for 10 min. Cells were then incubated in a blocking buffer (1% BSA and 0.1% Triton X-100) for 1 h at 4°C. The cells were then incubated with primary antibodies at 4 °C overnight for immunofluorescence (IF). The next day, the cells were incubated with the appropriate biotinylated secondary antibodies for 1 h, and then incubated with Alex Fluor 488 (Molecular Probes, Eugene) for 1 h. Finally, cells were counterstained with DAPI for 15 min. Coverslips were examined by confocal microscope (Carl Zeiss, Jena, Germany).

### Animal experiments

This experiment was conducted in accordance with the humane treatment of animals under institutional guidelines approved by the Ethical Committee of Soochow University. Five-week-old nude (BALB/c) mice were used in the experiments (Animal Experiment Center of Soochow University, Suzhou, China). RKO and HT29 cells (5 × 10^7^ cells/0.1 mL medium/mouse) were injected into the mice subcutaneously to establish the mouse models. Xenografts were allowed to grow to about 100 mm^3^ and randomly divided into four groups (*n* = 6 in each group) as follows: control group, IR group, SN50 group (15 mg/kg), and SN50 + IR group. During irradiation, the head and heart of the nude mice should be protected from irradiation and the irradiation area should be adjusted to accommodate the subcutaneous graft tumor. The nude mice were irradiated with X-RAD 320 iX X-ray irradiation instrument, the dose rate was 1.5 Gy/min, the distance from the skin source was 50 cm, and the total dose was 20 Gy. The nude mice were irradiated twice a week apart and SN50 + IR group was injected with SN50 before IR. At the end of the experiments, the tumor tissues of mice were taken for immunohistochemistry.

### Immunohistochemical staining

Immunostaining was performed using the Vectastain ABC kit (Vector) according to the manufacturer’s instructions. First, the slides were dewaxed, rehydrated, and treated with a citric acid solution in preparation for immunohistochemical studies. After 3% hydrogen peroxide solution blocked endogenous peroxidase activity, the slides were incubated in the blocking solution (PBS, 3% bovine serum albumin), and then incubated with the primary antibodies. After 24 h, HRP and DAB solutions were applied according to the manufacturer’s instructions. The sections were counterstained with hematoxylin for nuclear staining. The slices were then dehydrated and dried after being rinsed separately in acidic solution and alkaline solution. Finally, the slides were covered in resin and observed with a microscope at a magnification of 400.

### Statistical analysis

Data were expressed as mean ± S.E.M. At least three independent experiments were performed. Student’s *t*-test was used for statistical analysis. *P* < 0.05 was denoted statistically significant.

## Supplementary information

s1

STR profiling
