# Supplementary figures and images for: NF-κB is involved in the regulation of autophagy in mutant p53 cells in response to ionizing radiation
Source: Cell Death Discov. 2021 Jun 25;7:159. doi: 10.1038/s41420-021-00533-w (PMC8257568; doi:10.1038/s41420-021-00533-w)

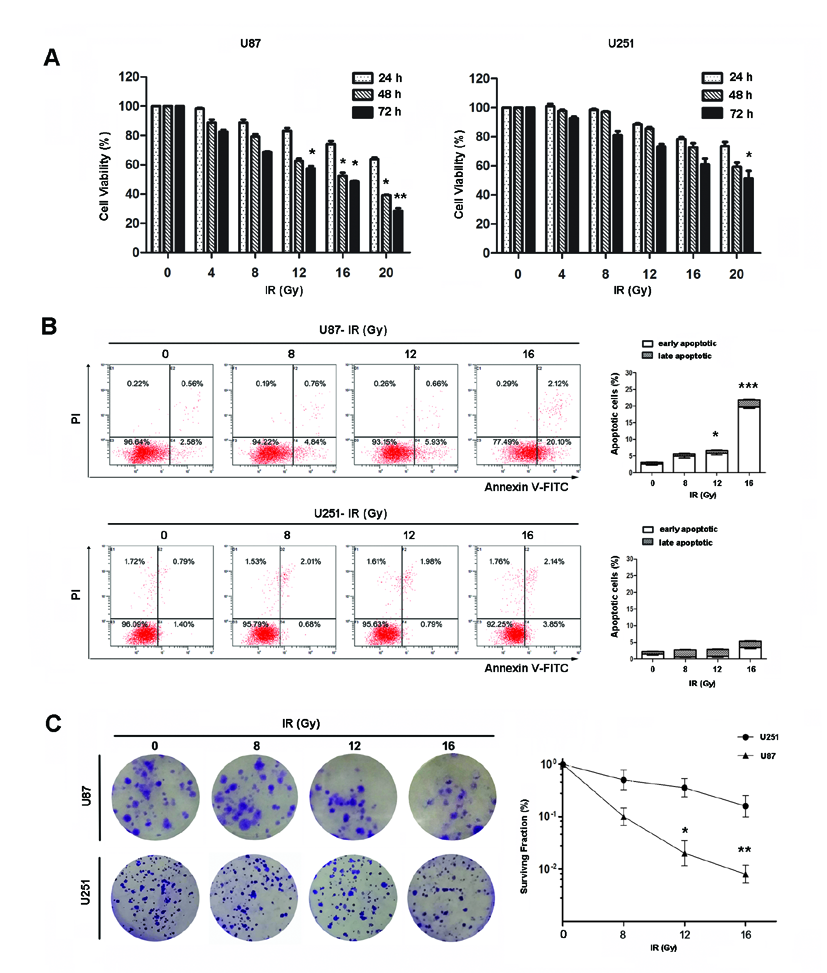

Supplement: Supplementary file 1 — s1 [file 41420_2021_533_MOESM1_ESM.tif]
